# Entomotherapy as an alternative treatment for diseases due to Gram-negative bacteria in Burkina Faso

**DOI:** 10.1038/s41598-023-50622-2

**Published:** 2024-01-02

**Authors:** Mamadou Ouango, Hama Cissé, Rahim Romba, Samuel Fogné Drabo, Rasmané Semdé, Aly Savadogo, Olivier Gnankiné

**Affiliations:** 1 Laboratoire d’Entomologie Fondamentale et Appliquée, Université Joseph KI ZERBO, 03 BP 7021 Ouagadougou, Burkina Faso; 2 Laboratoire de Biochimie et Immunologie Appliquées, Université Joseph KI ZERBO, 03 BP 7021 Ouagadougou, Burkina Faso; 3 Laboratoire du Développement du Médicament, Centre de Formation, de Recherche et d’Expertise en Sciences du Médicament, Université Joseph KI ZERBO, 03 BP 7021 Ouagadougou, Burkina Faso

**Keywords:** Microbiology, Medical research

## Abstract

Insects are known for their harmful effects. However, they also benefit humans, animals, plants, and ecosystems. Its beneficial uses include entomophagy and entomotherapy. This study aimed to evaluate the antibacterial activity of insect extracts against Gram-negative bacteria. Antibacterial activities of thirteen crude extracts of medicinal insects were tested against twelve Gram-negative bacteria by diffusion on agar. Imipenem was used as an antibiotic for positive control. The thirteen extracts acted differently against certain Gram-negative bacteria. The largest inhibition diameter was for extracts of *Cirina butyrospermi* and *Mylabris variabilis* against *Pseudomonas aeruginosa* ATCC27853 and *Salmonella enteritidis* ATCC13076, respectively. The diameters of inhibition obtained using imipenem against these same bacterial strains were 13.0 ± 0.0 mm and 22 ± 1.0 mm, respectively. The lowest inhibition diameter (7.5 ± 0.0 mm) was obtained using *Anopheles gambiae* extract against *Salmonella* Typhimurium ATCC14028. Imipenem was active on all strains tested. The highest values of the index multi-resistance to insect’s extracts were reported for *Pseudomonas aeruginosa* ATCC9027 and *Serratia odorifera* 652411. Overall, the results of this study confirmed the antibacterial activities of insects used by traditional health practitioners to treat different pathologies. Entomotherapy could be an alternative treatment for certain infectious pathologies caused by gram-negative bacteria.

## Introduction

Insects are the most numerous groups of living beings, with over 1.3 million described species^1^. These insects develop a wide range of peptides to colonize different ecosystems to defend themselves against invaders. Based on this observation, humans have become interested in using them to treat certain diseases^2–6^. The therapeutic potential of these insects is believed to be linked to the secretion of antimicrobial peptides. These antimicrobial peptides, which humans use, have antibacterial, antifungal, antiviral, and antiparasitic activities^7–10^. Research on antimicrobial peptides began in the 1980s with the discovery of drosomycin as the first antimicrobial peptide discovered in insects^11^. Research into antimicrobial peptides has recently increased to address bacterial multidrug resistance to commonly used antibiotics. Bacterial resistance to antibiotics is a major public health concern. According to the WHO, if nothing is done to find a palliative solution for antimicrobial resistance, it will cause 10 million deaths annually by 2050^12^.

However, Gram-negative bacteria are the most implicated in bacterial multidrug resistance due to the production of beta-lactamases (penicillinase, cephalosporinases, and carbapenemases). These Gram-negative bacteria are responsible for many serious infections, such as pneumonia, peritonitis, urinary tract infections, sepsis, kidney sepsis, wound infections, and meningitis. These infections are caused by *Acinetobacter, Enterobacter, Klebsiella, Proteus, Pseudomonas*, *E. coli,* and *Serratia*^13^. Thus, apart from infections, some are responsible for food poisoning and typhoid fevers, such as *Salmonella*, *Shigella*, *E. coli*, and *Pseudomonas* strains.

Despite their pathogenicity, some Gram-negative bacteria are exploited in food technology, medicine, and agriculture. Bacteria are responsible for the production of antimicrobial peptides (nisin, colicin, and natamycin), exopolysaccharides (dextrans, β-glucans, fructans, and levans), and enzymes (hydrolases, proteases, peptidase, and lipases)^14–16^. These compounds have biotechnological applications, such as texturing agents to modify the viscosity and elasticity of food products^15^. In recent years, these bacteria have been the center of interest of researchers because of their potential application in food bio-preservatives and their probiotic properties^14,15^. Certain Gram-negative bacteria such as *Gluconoacetobacter diazotrophicus, Acetobacter nitrogenifigens, Gluconoacetobacter sacchari, Gluconoacetobacter kombuchae, Acetobacter aceti*, and *Gluconobacter oxydans* are exploited specifically to produce alcohols and acetic acid. Certain strains of Salmonella and Escherichia coli are used as candidate adjuvants for sublingual allergy treatment vaccines to improve the clinical effectiveness of immunotherapy and allergens. However, the use of antibiotics as growth factors in food additives favors the rapid development of bacterial resistance to antibiotics.

Certain Gram-negative bacteria resist several available antibiotics and easily acquire antibiotic-resistance genes. Gram-negative bacteria are more difficult to eliminate. This means that Gram-positive and Gram-negative bacteria require different treatments. These carbapenemase and beta-lactamase-producing bacteria are common in West Africa^17^. However, the spread of Gram-negative bacteria producing carbapenemases and extended-spectrum beta-lactamases constitutes an “urgent” threat. Studies carried out by Sanou et al.^18^ have reported the presence of these in patients in health centers in Burkina Faso. Indeed, medicinal insects produce interesting antimicrobial peptides. In addition, when faced with an increase in bacterial resistance to antibiotics. Therefore, it is imperative to identify novel bioactive molecules. In Burkina Faso, *Apis mellifera*, *Cirina butyrospermi*, *Macrotermes belliccosus*, *Sceliphron* sp., *Periplaneta americana*, and many other insects have been used empirically in traditional medicine as medicinal insects^19^. The objective of this study was to evaluate the antimicrobial activity of medicinal insects collected from three phytogeographical areas of Burkina Faso against Gram-negative bacteria.

## Materials and Methods

### Sites of collection insects’

The insects were collected in three provinces namely Kadiogo (12°21′ 56.4''N; 1°32′2''W), *Houët* (11°7′ 55.36''N; 4°14′0.01'' W) and *Séno* (14°1′ 48''N; 0°1′48'' W), which belong to three different phytogeographic zones. We conducted a survey among traditional health practitioners at these sites to determine their knowledge of medicinal insects. These sites were chosen because of the cosmopolitan nature of traditional health practitioners.

### Technical collection and information on the insect collected in this study

Insects were collected mainly using threshing methods, swath nets, and mechanical sampling with laboratory tweezers in insect nests. The insects were then placed in alcohol jars to preserve them^20–22^. Thirteen medicinal insects were collected based on information provided by traditional health practitioners. These insects are listed and illustrated in Table 1. Once arrived at the laboratory, the collected insects were dried in an oven at a constant temperature of 25 °C until completely dry. This allows the collected samples to maintain their complete integrity while preventing their decomposition. The collection of these insects was conducted from July 2022 to September 2023, a period of 15 months. The number of each insect species collected varied significantly from one species to another. Indeed, we had for each type of insect specimen a quantity in dry weight of 20 g. Thus, for large insects such as *Periplanneta americana*, *Mylabris variabilis*, *Lytta* sp., *Kraussaria anguilifera*, *Acrida bicolor*, *Cirina butyrospermi*, and *Bunaea alcinoe*, the number of specimens was 45. On the other hand, for small insects such as *Macrotermes bellicosus*, *Odontotermes* sp., *Pachycondyla* sp., *Acheta domesticus*, and *Anopheles gambiae*, the number of individuals collected per specimen varied from a hundred to several hundred.

**Table 1:** Presentation of insects collected in this study.

Common name or local name	Scientific name	Stage of development	Picture
Grillon domestique (French) House cricket (English) *Sokɛɛrɛɛ* (*Dioula*) *Buglunvare* (Moore)	*Acheta domesticus* (Linnaeus, 1758)	Imago	
Moustique (French) Mosquito (English) *Soso* (*Dioula*) *Ruunga* or *rumsi* (Moore)	*Anopheles gambiae* (Giles, 1902)	Imago	
Abeillle (French) Bee (English) *Nyaaku* (*Fulfulde*) *Siinfu or Sii* (Moore)	*Apis mellifera* (Linnaeus, 1758)	Imago	
Chenille de caïlcédrat (French) African mahogany caterpillar (English) *Djalayiri-tumu* (*Dioula*) *Gouwerba Kuka* (*Moore*)	*Bunaea alcinoe* (Stoll, 1780)	Larva	
Chenille de karité (French) Caterpillar of Cirina butyrospermi (English) *Sii-tumu* (*Dioula*) *Gouwerba taanga* (*Moore*)	*Cirina butyrospermi* (Vuillet, 1911)	Larva	
Cantharide (French) *Blister beetle (English)* *Pusg-n-waag-ma* (*Moore*)	*Lytta sp*. (Fabricius, 1775)	Imago	
Termite (French) African mound building termite (English) *Kinkinba* (*Moore*)	*Macrotermes bellicosus* (Smeathman, 1781)	Imago	
*Mylabris* (French) *Banded blister beetle (English)*	*Mylabris variabilis* (Pallas, 1781)	Imago	
Termite (French) Fungus-growing termites (English) *Moogdo or Yao-bisi* (*Moore*)	*Odontotermes sp.* (Holmgren, 1912)	Imago	
Blatte (French) American cockroach (English) *ɲɛbɛrɛ* (*Dioula*) *Yalaare or takaluuta* (Fulfulde) *Yaalé* (*Moore*)	*Periplaneta americana* (Linnaeus, 1758)	Imago	
Fourmis (French) Panther ants (English) *Gũuri* (*Moore*)	*Pachychondyla sp*. (Smith, 1858)	Imago	
Criquet (French) Sahelian grasshopper (English) *Toon* (*Dioula*) *Suuré* (*Moore*)	*Kraussaria angulifera* (Krauss, 1877)	Imago	
Truxale (French) Long-headed grasshopper (English) *Toon* (*Dioula*) *Suuré* (*Moore*)	*Acrida bicolor* (Thunberg, 1815)	Imago	

### Preparation of the insects for extraction

After the drying step, the insects were finely ground using a ceramic laboratory mortar. The insect powder was packaged in Falcon tubes (CONICAL BOTTOM CELLSTAR® STERILE) and stored in the oven at 37 °C for future use.

### Extraction of crude extracts from insects collected

The extraction of crude extracts from insects was performed according to the method described by Dah-Nouvlessounon et al.^23^ and readapted. Thus, aqueous extraction (crude extracts) was performed using sterile ultrapure Milli-Q water. For the realization, 4 g of each insect powder was macerated in 10 mL of the extraction solution for 12 h at 25 °C under magnetic stirring. The macerates were centrifuged at 3,000 rpm for 10 min at 4 °C using a JOUAN BR4 refrigerated centrifuge. After centrifugation, the supernatants were collected in Eppendorf tubes and kept cool at 4 °C. After collecting the supernatants, and the extraction solvents were evaporated to dryness in an oven at 45 °C until a dry extract of constant mass was obtained for the evaluation of extraction yield. The residues obtained were kept at 4 °C until the antimicrobial tests were performed.

### Extraction yield

The extraction yield was determined by the ratio between the mass of the powdered insect after extraction and the mass of their ground material at the start according to the following formula.

$$\mathrm{Extraction \;yield}=\frac{\mathrm{Mass \;of \;dry \;extract}}{\mathrm{Initial \;mass \;of \;test \;portion}}\mathrm{X }100$$

### Gram-negative bacteria used for antimicrobial testing

Antimicrobial activity tests were carried out against twenty-two microbial germs including twelve Gram-negative bacteria (Table 2). Microbial strains used in this study are based on several criteria: these strains are commonly of hospital and food origin, for their high incriminations in pathologies in animals and humans, and these strains are chosen regarding their natural resistance to various types of antimicrobial agents.

**Table 2:** Information on the Gram-negative bacteria tested.

Microorganism	Species	Reference
Gram-negative bacteria	*Escherichia coli*	652654
	*Escherichia coli*	ATCC25922
	*Escherichia coli*	ATCC8739
	*Klebsiella pneumoniae*	203
	*Klebsiella pneumoniae*	ATCC13883
	*Providencia rettgeri*	652655
	*Pseudomonas aeruginosa*	ATCC9027
	*Pseudomonas aeruginosa*	ATCC27853
	*Salmonella abony*	NCTC6017
	*Salmonella enteritidis*	ATCC13076
	*Salmonella* Typhimurium	ATCC14028
	*Serratia odorifera*	652411

### Antimicrobial activity testing of crude extracts from insects

The antimicrobial activity of crude extracts from insects was tested according to the agar diffusion method described by Kirby-Bauer following the guidelines of the Clinical Laboratory Standards Institute^24^. The microbial inocula was prepared from young colonies aged from 16 to 18 h diluted in test tubes containing physiological saline. All microbial suspensions obtained were adjusted to a turbidity of 0.5 MacFarlant. This standard turbidity of 0.5 McFarland corresponds approximately to a culture density of 1.5 × 10^8^ cells/mL. For the preparation of discs containing crude extracts from insects, blank and sterile test antibiogram discs (MASTDISCS® AST) of 6 mm of diameter were used. These discs were impregnated by solutions of crude extracts from insects contained in Eppendorf tubes for 10 min. As for carrying out the antibiogram, Mueller–Hinton agar was used. Petri dishes containing the agar were inoculated by swabbing with the culture of different bacterial strains. The inoculated agars were dried near a Bunsen burner for 5 min before receiving discs impregnated with crude extracts from insects. Imipenem was used as positive control, and blank and sterile test antibiogram discs impregnated in DMSO without extract were used as negative control. After depositing discs (six antibiotic discs were used for each box, except that receiving the positive control which contained seven discs), Petri dishes were left at room temperature during 15 min to allow the diffusion of extracts and incubated at 37 °C during 24 h. The inhibition diameters materialized by a clear halo around the disc were measured using a BioNumerical ruler (MICROBIAL DATA ANALYSIS SOFT WARE).

### Determination of index multi-resistance to crude extracts from insects

The index of multi-resistance to extracts (IMRE) of crude extracts from insects was determined according to Das et al*.*^25^. IMRE was calculated using the following formula:

$${\text{IMRE}}=\frac{\mathrm{Number \;of \;ineffective \;extracts \;on \;microbial \;strain}}{\mathrm{Total \;number \;of \;extracts \;tested \;on \;microbial \;strain}}$$

### Activity coefficient of crude extracts from insects

The activity coefficient (A) of crude extracts from insects collected against the bacterial strains tested was calculated using the following formula:

$$A=\frac{\pi {Z}^{2}}{4{\text{Q}}}$$

Q: quantity of insect extract (μL); Z: inhibition diameter including the diameter of the disc (cm).

### Data processing and statistical analyses

Results were expressed as a mean number followed by standard deviation (M ± SD) and subject to Student's t-test using R. The significance threshold was 5%. XLSTAT-2019 software was used for principal component analysis (PCA). PCA was used to explore the correlation between the activities of crude extracts from insects collected and the different Gram-negative bacteria.

## Results and discussion

### Yield values of extractions of crude extracts from insects

**Figure 1:**
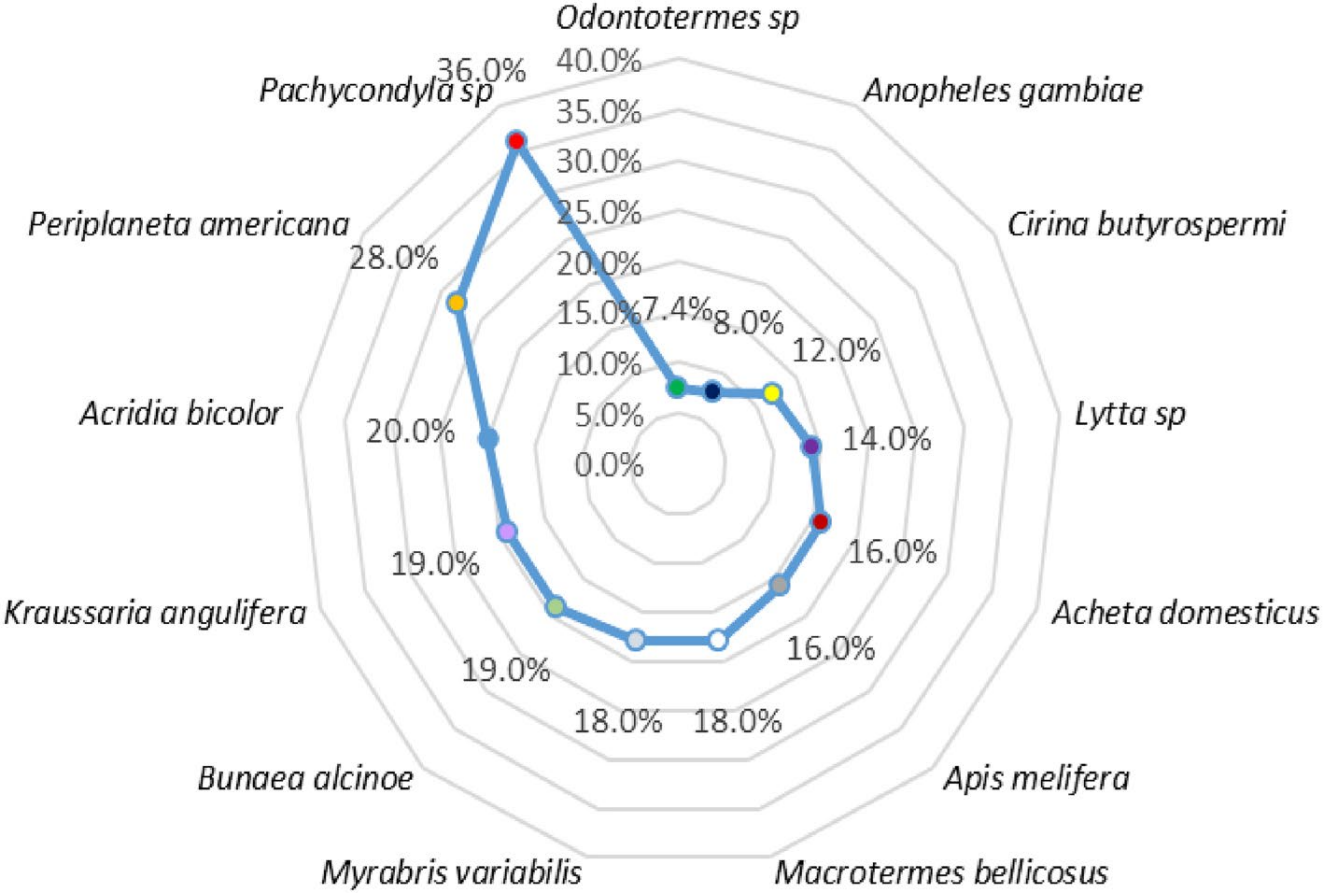
Extraction yields of the insects used.

The different yields of the compounds in the crude extracts obtained after extraction are recorded in Fig. 1. These yields vary with the species of insects. The highest yields were obtained with (36.0%) *Pachychondyla sp*., (28.0%) *Periplaneta americana*, and (20.0%) *Acrida bicolor* and weak yields with (8.0%) *Anopheles gambiae* and (7.4%) *Odontotermes sp*. The former contains a more water-soluble matter than the latter. Thus, several factors could strongly influence the extraction yield. Among these factors are drying time, particle size of the ground material, nature of the solvent used, mass-volume ratio of the ground’s solvent (m/v), maceration time, and stirring speed.

### Antimicrobial activity of crude insects extracts

**Figure 2:**
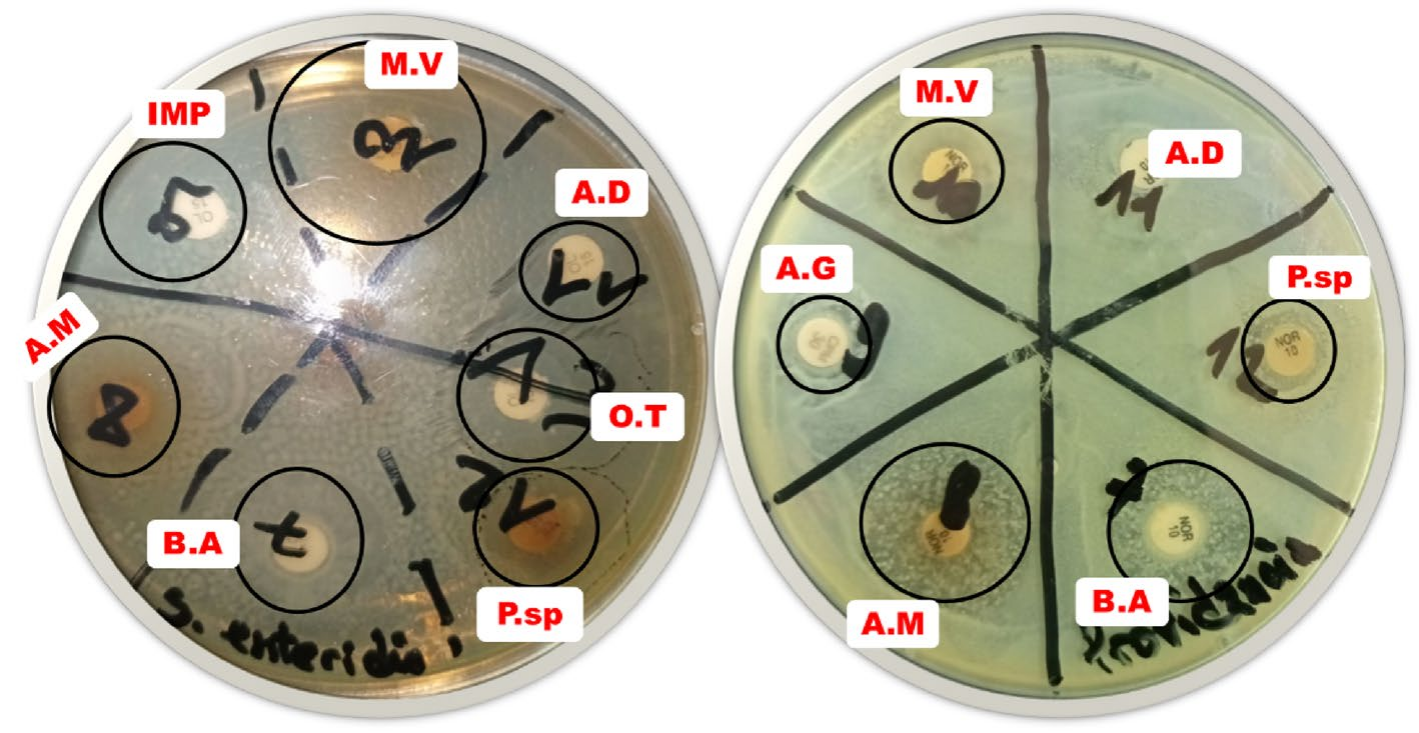
Antimicrobial activity of insect extracts on *Salmonella enteritidis* and *Providentia rettgeri*. B.A: *Bunea alcinoe*; P.sp.: *Pachychondyla sp*.; A.D: *Acheta domesticus ;* M.V : *Mylabris variabilis ;* IMP: Imipenem ; A.G: *Anopheles gambiae;* A.M : *Apis mellifera ;* O.T : *Odontotermes sp.*

The crude extracts from insects tested inhibited the growth of the bacterial strains, as shown in Fig. 2 and Table 3. The inhibition diameters varied depending on the strain and the extract used. All thirteen crude extracts inhibited the growth of some bacterial.

**Table 3:** Inhibition diameters of insect extracts against Gram-negative bacteria. Values in the same column with different superscript letters are significantly different (p < 0.05) to the Student's t-test.

Gram-negative bacteria strains	*Acheta domesticus*	*Anopheles gambiae*	*Apis melifera*	*Bunaea alcinoe*	*Cirina butyrospermi*	*Lytta sp.*	*Macrotermes bellicosus*	Imipenem
*Escherichia coli* 652654	9.0 ± 0.0^b^	10.5 ± 0.5^d^	0.0 ± 0.0^a^	0.0 ± 0.0^a^	9.5 ± 0.05^b^	12.0 ± 0.0^de^	0.0 ± 0.0^a^	20.0 ± 0.0^d^
*Escherichia coli* ATCC25922	16.5 ± 0.5^e^	10 ± 0.0^d^	9.0 ± 0.0^b^	0.0 ± 0.0^a^	11.0 ± 0.0^c^	10.0 ± 0.0^bc^	0.0 ± 0.0^a^	22.0 ± 0.0^e^
*Escherichia coli* ATCC8739	0.0 ± 0.0^a^	0.0 ± 0.0^a^	0.0 ± 0.0^a^	0.0 ± 0.0^a^	0.0 ± 0.0^a^	13.0 ± 0.0^e^	9.5 ± 0.5^b^	17.0 ± 0.0^c^
*Klebsiella pneumoniae* 203	15.5 ± 0.5^de^	0.0 ± 0.0^a^	15.0 ± 1.0^ cd^	0.0 ± 0.0^a^	0.0 ± 0.0^a^	11.0 ± 0.0^ cd^	0.0 ± 0.0^a^	15.0 ± 0.0^b^
*Klebsiella pneumoniae* ATCC13883	18.5 ± 1.5f	14.0 ± 0.0^e^	0.0 ± 0.0^a^	10.0 ± 0.0^b^	12 ± 1.0^ cd^	0.0 ± 0.0^a^	12.0 ± 0.0^c^	12.0 ± 0.0^a^
*Providencia rettgeri* 652655	0.0 ± 0.0^a^	10.5 ± 0.5^d^	22.5 ± 0.5^e^	19.5 ± 0.5^c^	8.5 ± 0.5^b^	13.5 ± 1.5^e^	0.0 ± 0.0^a^	15.0 ± 0.0^b^
*Pseudomonas aeruginosa* ATCC9027	0.0 ± 0.0^a^	0.0 ± 0.0^a^	0.0 ± 0.0^a^	20.0 ± 0.0^ cd^	26.0 ± 0.5f.	0.0 ± 0.0 ^a^	0.0 ± 0.0^a^	15.5 ± 0.5^e^
*Pseudomonas aeruginosa* ATCC27853	12.5 ± 0.0^c^	9.0 ± 0.0^c^	14.5 ± 0.5^c^	0.0 ± 0.0^a^	30.0 ± 0.0^ h^	0.0 ± 0.0^a^	13.5 ± 0.5^d^	13.0 ± 0.0^d^
*Salmonella abony* NCTC6017	14.0 ± 1.0^d^	15.0 ± 0.0^e^	8.0 ± 0.0^b^	10.0 ± 0.0^b^	14.0 ± 0.0f.	12.0 ± 0.0^de^	9.0 ± 0.0^b^	28.0 ± 1.0^ g^
*Salmonella enteritidis* ATCC13076	14.0 ± 1.0^d^	0.0 ± 0.0^a^	16.0 ± 0.0^d^	20.5 ± 0.5^d^	18.5 ± 0.5^ g^	10.0 ± 0.0^bc^	11.0 ± 1.0^c^	22.0 ± 1.0^e^
*Serratia odorifera* 652411	0.0 ± 0.0^a^	15.0 ± 1.0^e^	0.0 ± 0.0^a^	0.0 ± 0.0^a^	0.0 ± 0.0^a^	12.5 ± 0.5^de^	0.0 ± 0.0^a^	13.0 ± 0.0^a^
*Salmonella* Typhimurium ATCC14028	10.0 ± 0.0^b^	7.5 ± 0.0^b^	0.0 ± 0.0^a^	0.0 ± 0.0^a^	12.5 ± 0.5^d^	9.0 ± 1.0^b^	8.5 ± 0.5^b^	19.5 ± 0.5^d^

Gram-negative bacteria strains	*Myrabris variabilis*	*Odontotermes sp.*	*Periplaneta americana*	*Pachycondyla* sp.	*Kraussaria angulifera*	*Acridia bicolor*	Imipenem	Discs with DMSO
*Escherichia coli 652654*	10.0 ± 0.0^b^	0.0 ± 0.0^a^	9.0 ± 0.0^b^	14.5 ± 0.5^e^	0.0 ± 0.0^a^	0.0 ± 0.0^a^	20.0 ± 0.0^d^	Inhibition diameter of DMSO-impregnated discs used as a positive control (For all this study) were 00.0 ± 0.0
*Escherichia coli ATCC25922*	0.0 ± 0.0^a^	0.0 ± 0.0^a^	12.0 ± 0.0^d^	14.0 ± 0.0^de^	0.0 ± 0.0^a^	0.0 ± 0.0^a^	22.0 ± 0.0^e^	
*Escherichia coli ATCC8739*	14.5 ± 0.5^e^	0.0 ± 0.0^a^	13.0 ± 1.0^d^	13.0 ± 0.0^ cd^	8.0 ± 0.0^b^	17.5 ± 1.5^c^	17.0 ± 0.0^c^	
*Klebsiella pneumoniae 203*	0.0 ± 0.0^a^	0.0 ± 0.0^a^	8.0 ± 0.0^b^	0.0 ± 0.0^a^	0.0 ± 0.0^a^	0.0 ± 0.0^a^	15.0 ± 0.0^b^	
*Klebsiella pneumoniae ATCC13883*	0.0 ± 0.0^a^	19.5 ± 0.5^d^	15.0 ± 1.0^e^	15.0 ± 0.0^e^	0.0 ± 0.0^a^	18.5 ± 0.5^c^	12.0 ± 0.0^a^	
*Providencia rettgeri 652655*	15.0 ± 0.0f	0.0 ± 0.0^a^	0.0 ± 0.0^a^	12.5 ± 0.5^c^	0.0 ± 0.0^a^	0.0 ± 0.0^a^	15 ± 0.0^b^	
*Pseudomonas aeruginosa* ATCC 9027	0.0 ± 0.0^a^	0.0 ± 0.0^a^	0.0 ± 0.0^a^	0.0 ± 0.0^a^	0.0 ± 0.0^a^	0.0 ± 0.0^a^	15.5 ± 0.5^e^	
*Pseudomonas aeruginosa* ATCC27853	14.5 ± 0.5^e^	9.0 ± 0.0^b^	10.5 ± 0.5^c^	10.0 ± 0.0^b^	15.0 ± 1.0^c^	16.5 ± 1.5^bc^	13.0 ± 0.0^d^	
*Salmonella abony* NCTC6017	12.0 ± 0.0^d^	0.0 ± 0.0^a^	0.0 ± 0.0^a^	11.0 ± 1.0^b^	12.5 ± 0.5^c^	15.0 ± 0.0^b^	28.0 ± 1.0^ g^	
*Salmonella enteritidis* ATCC1307*6*	30.0 ± 0.0^ g^	18.0 ± 0.0^c^	0.0 ± 0.0^a^	13.0 ± 1.0^ cd^	16.0 ± 1.0^d^	14.5 ± 1.5^b^	22.0 ± 1.0^e^	
*Serratia odorifera* 652411	0.0 ± 0.0^a^	0.0 ± 0.0^a^	0.0 ± 0.0^a^	0.0 ± 0.0^a^	0.0 ± 0.0^a^	0.0 ± 0.0^a^	13.0 ± 0.0^a^	
*Salmonella* Typhimurium ATCC14028	11.0 ± 0.0^c^	0.0 ± 0.0^a^	17.0 ± 0.0f	13.0 ± 0.0^ cd^	20.0 ± 0.0^e^	23.0 ± 1.0^d^	19.5 ± 0.5^d^	

The crude extract of *Acheta domesticus* was active on eight and twelve Gram-negative bacteria. The highest inhibition diameter was 18.5 ± 1.5 mm against *Klebsiella pneumoniae* ATCC13883, whereas the lowest was 09.0 ± 0.0 mm against *Escherichia coli* 652654. The crude extract of *Acheta domesticus* did not affect *Escherichia coli* ATCC8739, *Providencia rettgeri* 652655, and *Serratia odorifera* 652411. In Latin America, *Acheta domesticus* has been used in treating tract infections in Brazil and as an antidiuretic against urinary retention in Mexico^26–28^. The bioactive molecule contained in *Acheta domesticus* is phenoloxidase^29^.

The crude extract of *Anopheles gambiae* was active on eight Gram-positive bacteria, with an inhibition percentage of 66.66%. The highest inhibition diameter was 15.0 ± 1.0 mm and the lowest was 07.5 ± 0.0 mm against *Salmonella abony* NCTC6017 and *Salmonella* Typhimurium ATCC14028, respectively. *Anopheles gambiae* extract had no inhibitory effect on *Escherichia coli* ATCC8739, *Klebsiella pneumoniae* 203, *Pseudomonas aeruginosa* ATCC27853, and *Salmonella enteritidis* ATCC13076. The inhibitory effect of *Anopheles gambiae* extract may be linked to its richness in different proteins^30^. Indeed, AMPs such as cecropins are found in this insect^11^.

*Apis mellifera* crude extract was active on six of the bacterial strains (50% inhibition), with the greatest inhibition diameter of 22.5 ± 0.5 mm (*Providencia rettgeri* 652655) and the lowest inhibition diameter of 08.0 ± 0.0 mm (*Salmonella abony* NCTC6017). This crude extract had no inhibitory effect on *Escherichia coli* 652654, *Escherichia coli* ATCC8739, *Klebsiella pneumoniae* ATCC13883, *Pseudomonas aeruginosa* ATCC9027, *Serratia odorifera* 652411, and *Salmonella* Typhimurium ATCC14028. The recorded antimicrobial activity may be due to the presence of melittin in the bee venom. Lupoli^31^ and Marques et al.^32^ reported the presence of inhibitory molecules, such as melittin, the main peptide in bee venom.

Active on four of the twelve Gram-negative bacteria (that is, 33.33% inhibition rate), the extract of *Bunaea alcinoe* had strong inhibitory activity against *Salmonella enteritidis* ATCC13076 (20.5 ± 0.5 mm) and weak inhibitory activity against *Klebsiella pneumoniae* ATCC13883 and *Salmonella abony* NCTC6017 (10.0 ± 0.0 mm). The extract did not affect the three Gram-negative bacteria strains tested as *Klebsiella pneumoniae* ATCC13883, *Pseudomonas aeruginosa* ATCC27853, *Serratia odorifera* 652411, and *Salmonella* Typhimurium ATCC14028. *Bunaea alcinoe* extract has antibacterial, antitumor, and antidiuretic effects because tannin contains^33,34^.

Thus, a 75% inhibition rate of the bacterial strains tested (9/12) was observed with *Cirina butyrospermum* extract. The lowest and highest diameters of inhibition were respectively 08.5 ± 0.5 mm (*Providencia rettgeri* 652655) and 30.0 ± 0.5 mm (*Pseudomonas aeruginosa* ATCC9027). Shea caterpillars are known to be rich in proteins, accounting for more than 60% of the total^35,36^. Some of these proteins have antibacterial activity. However, this extract was not active against *Escherichia coli* ATCC8739, *Klebsiella pneumoniae* ATCC13883, and *Serratia odorifera* 652411.

The *Lytta* sp. extract also inhibited 75% of the bacterial strains tested. Its maximum inhibition diameter (13.5 ± 1.5 mm) was recorded against *Providencia rettgeri* 652655 and the lowest inhibition diameter (09.0 ± 1.0 mm) with *Salmonella* Typhimurium ATCC14028. This meloid did not inhibit the growth of *Klebsiella pneumoniae* and the two strains of *Pseudomonas aeruginosa* tested. This inhibitory activity was attributed to cantharidin. Cantharidin is a bioactive molecule concentrated on the genital glands of insects in the genus *Lytta*, which belongs to the meloid family^31^.

Six of twelve bacterial strains were inhibited by the *Macrotermes bellicosus* extract (i.e., an inhibition rate of 50%). *Pseudomonas aeruginosa* ATCC27853 strain was the most sensitive (13.5 ± 0.5 mm), and *Salmonella* Typhimurium ATCC14028 was the least sensitive strain (08.5 ± 0.5 mm). This extract was found to be effective against the different *Salmonella* strains tested. This result is consistent with that reported by Afolejan et al.^37^. These authors revealed the inhibitory action of *Macrotermes bellicosus* soldier extracts on different *Salmonella* strains. Hydroquinone and acid gluconic acid are the two molecules with antibacterial activity in *Macrotermes* extracts^6^. However, some strains showed resistance to the crude extracts of this insect. These were *Escherichia coli* 652654, *Escherichia coli* ATCC25922, *Klebsiella pneumoniae* 203, *Providencia rettgeri* 652655, *Pseudomonas aeruginosa* ATCC9027, and *Serratia odorifera* 652411.

*Mylabris variabilis* extract inhibited 58.33% of the bacterial strains tested. The inhibitory activity was more remarkable against *Salmonella enteritidis* ATCC13076 (30 ± 0.0 mm), unlike *Escherichia coli* 652654 (10.0 ± 0.0 mm). In contrast, for *Escherichia coli* ATCC25922, the two strains of *Klebsiella* tested, *Pseudomonas aeruginosa* ATCC9027, and *Serratia odorifera* 652411, the extract had no inhibitory effect. *Mylabris* extract contains inhibitory molecules such as cantharidin, which is strongly produced by the *Mylabris* genus^31,38^.

The extract of *Odontotermes* sp. was only active against the three bacterial strains (25% inhibition). The highest inhibition diameter (19.5 ± 0.5 mm) was reported for *Klebsiella pneumoniae* ATCC13883, and the lowest inhibition diameter (09.0 ± 0.0 mm) was reported for *Pseudomonas aeruginosa* ATCC27853. Gram-negative bacteria resistant to this extract are the three *Escherichia coli* strains tested *Klebsiella pneumoniae* 203, *Providencia rettgeri* 652655, *Pseudomonas aeruginosa* ATCC9027, *Salmonella abony* NCTC6017, *Serratia odorifera* 652411, and *Salmonella* Typhimurium ATCC14028. The antimicrobial activity could be due to the bioactive molecules produced by the actinomycetes that these insects harbor^39^.

The inhibition rate assigned to *Periplaneta americana* was 58.33% (seven of twelve bacteria tested). *Salmonella* Typhimurium ATCC14028 was the most sensitive strain to extracts of *Periplaneta americana*, and *Klebsiella pneumoniae* 203 and the least sensitive strain with inhibition diameters of 17.0 ± 0.0 mm, and 08.0 ± 0.0 mm, respectively. However, five strains were resistant to the *Periplaneta americana* extract. These include *Providencia rettgeri* 652655, *Pseudomonas* aeruginosa, *Salmonella abony* NCTC6017; *Salmonella enteritidis* ATCC13076, and *Serratia odorifera* 652411. The antibacterial activity could be attributed to the AMPs in this insect. Indeed, a study conducted in 2016 by Kim et al.^40^ made it possible to isolate twelve AMPs with strong antibacterial activity. Basserie et al.^41^ and Ali et al.^42^ were also identified AMPs of *Periplaneta americana*.

The extract of *Pachycondyla* sp. revealed inhibitory activity against nine of the twelve strains tested (75% inhibition). The diameter of inhibition against *Klebsiella pneumoniae* ATCC13883 was the highest (15.0 ± 0.0 mm). However, the diameter of inhibition reported against *Pseudomonas aeruginosa* ATCC27853 was the lowest (10.0 ± 0.0 mm). Santos et al.^43^ reported that extracts of the *Pachychodyla* genus contained broad-spectrum inhibitory molecules that act against Gram-positive and Gram-negative bacteria.

For the test with *Kraussaria angulifera* extract, antibacterial activity was reported on five of the twelve bacterial strains tested, with the highest diameter of inhibition (20.0 ± 0.0 mm) against *Salmonella* Typhimurium ATCC14028 and the lowest diameter of inhibition (08.0 ± 0.0 mm) against *Escherichia coli* ATCC8739. The strains that were not sensitive to the *Orthoptera* extract were *Escherichia coli* 652654, *Escherichia coli* ATCC25922, and two strains of *Klebsiella pneumoniae* ATCC13883, *Providencia rettgeri* 652655, *Pseudomonas aeruginosa* ATCC9027, and *Serratia odorifera* 652411. Locusts contain excessive amounts of protein, fats, and essential fatty acids. Some of these proteins have inhibitory activities against certain bacteria^44–46^.

*Acrida bicolor* extract inhibited the growth of 6 bacterial strains (50% inhibition). The highest inhibition diameter (23.0 ± 1.0 mm) has been reported against *Salmonella* Typhimurium ATCC 4028. The lowest inhibition diameter of 14.5 ± 1.5 mm was obtained against *Salmonella enteritidis* ATCC13076. This insect extract did not inhibit the growth of *Escherichia coli* 652654*, Escherichia coli* ATCC25922*, Klebsiella pneumoniae* 203*, Providencia rettgeri* 652655*, Pseudomonas aeruginosa* ATCC 9027*, and Serratia odorifera* 652411. Bioactive molecules from grasshoppers are little known^27^. However, in Sudan, locusts and grasshoppers are used to treat stomach problems and jaundice, these potentialities could come from the plants that these insects consume^47^. Indeed, substances from plants of the *carnolidae* family, calotropin and calactin, have been found in certain locusts, such as *Poekilocerus bufonius* of the *Pyrgomorphidae* family^31^.

### Effectiveness of crude extracts insects compared to imipenem

For the three bacterial strains, the inhibition diameters reported with imipenem were greater than those of the insect extracts (Table 4), *Escherichia coli* 652654; *Escherichia coli* ATCC25922 and *Salmonella abony* NCTC6017. For the other nine bacterial strains, the inhibition diameters reported with imipenem were smaller than those of some insect extracts. The lowest difference in inhibition diameter (diameter reported with insect extract—diameter reported with imipenem) reported in the latter case was 0.5 mm reported with *Acridia bicolor* against *Escherichia coli* ATCC8739 and with *Acheta domesticus* against *Klebsiella pneumoniae* 203. The highest difference in inhibition diameter was 17 mm, as reported for *Cirina butyrospermi* against *Pseudomonas aeruginosa* ATCC27853. Table 4 shows the insect extracts for which the inhibition diameters were greater than those of imipenem against the bacterial strains tested.

**Table 4:** Insect extracts with higher inhibition than imipenem.

Gram-negative bacteria strains	Insect extracts with a diameter larger than that of imipenem	Difference in mm
*Escherichia coli* ATCC8739	*Acridia bicolor*	0.5 mm
*Klebsiella pneumoniae* 203	*Acheta domesticus*	0.5 mm
*Klebsiella pneumoniae* ATCC13883	*Acheta domesticus*	6.5 mm
	*Anopheles gambiae*	2 mm
	*Odontotermes sp.*	7.5 mm
	*Periplaneta americana*	3 mm
	*Pachycondyla sp*	3 mm
	*Acridia bicolor*	6.5 mm
*Providencia rettgeri* 652655	*Apis melifera*	7.5 mm
	*Bunaea alcinoe*	4.5 mm
*Pseudomonas aeruginosa ATCC9027*	*Bunaea alcinoe*	4.5 mm
	*Cirina butyrospermi*	10.5 mm
*Pseudomonas aeruginosa ATCC27853*	*Apis melifera*	1.5 mm
	*Cirina butyrospermi*	17 mm
	*Macrotermes bellicosus*	0.5 mm
	*Myrabris variabilis*	1.5 mm
	*Kraussaria angulifera*	2 mm
	*Acridia bicolor*	3.5 mm
*Serratia odolifera* 652411	*Anopheles gambiae*	2 mm
*Salmonella enteritidis* ATCC13076	*Myrabris variabilis*	8 mm
*Salmonella* Typhimurium ATCC14028	*Kraussaria angulifera*	0.5 mm
	*Acridia bicolor*	3.5 mm

### Index of multi-resistance to crude extracts from insects and their activity coefficient

The different indices of the Gram-negative bacteria tested with insect extracts are given in Table 5. Thus, 3 (25%) of the bacterial strains had an index of multi-resistance to insect’s extracts (IMRE) < 0.2 and 9(75%) have an IMRE > 0.2. For bacterial strains with an IMRE < 0.2, i.e., *Pseudomonas aeruginosa* ATCC27853, *Salmonella abony* NCTC6017, and *Salmonella enteritidis* ATCC13076, insect extracts could be used to effectively inhibit their development. As a result, these extracts can be used as. For the different extracts in which bacterial growth was inhibited, the activity coefficient was between 0.02 cm^2^/µL and 0.35 cm^2^/µL (Table 5). The best inhibitory actions were recorded with extracts of *Cirina butyrospermi* and *Myrabris variabilis* against *Pseudomonas aeruginosa* ATCC27853 and *Salmonella enteritidis* ATCC13076, respectively.

**Table 5:** Activity coefficient and indes of multi-resistance of crude extracts from insects.

Insects species	Gram-negative bacteria											
	*Pseudomonas aeruginosa* ATCC9027	*Pseudomonas aeruginosa* ATCC27853	*Salmonella abony* NCTC6017	*Salmonella enteritidis* ATCC13076	*Serratia odorifera* 652411	*Salmonella* Typhimurium ATCC14028	*E. coli* 652654	*E. coli* ATCC25922	*E. coli* ATCC8739	*Klebsiella pneumoniae* 203	*Klebsiella pneumoniae* ATCC13883	*Providencia rettgeri* 652655
	Activity coefficient (A) of insect extracts against bacterial strains in cm^2^/µL											
*Acheta domesticus*	0	0.06	0.08	0.08	0	0.04	0.03	0.11	0	0.09	0.13	0
*Anopheles gambiae*	0	0.03	0.09	0	0.09	0.02	0.04	0.04	0	0	0.08	0.04
*Apis melifera*	0	0.08	0.03	0.1	0	0	0	0.03	0	0.09	0	0.20
*Bunaea alcinoe*	0.16	0	0.04	0.16	0	0	0	0	0	0	0.04	0.15
*Cirina butyrospermi*	0.27	0.35	0.08	0.13	0	0.06	0.04	0.05	0	0	0.06	0.03
*Lytta sp.*	0	0	0.06	0.04	0.06	0.03	0.06	0.04	0.07	0.05	0	0.07
*Macrotermes bellicosus*	0	0.07	0.03	0.05	0	0.03	0	0	0.04	0	0.06	0
*Myrabris variabilis*	0	0.08	0.06	0.35	0	0.05	0.04	0	0.08	0	0	0.09
*Odontotermes sp.*	0	0.03	0	0.13	0	0	0	0	0	0	0.15	0
*Periplaneta americana*	0	0.04	0	0	0	0.11	0.03	0.06	0.07	0.03	0.09	0
*Pachycondyla sp.*	0	0.04	0.05	0.07	0	0.07	0.09	0.08	0.07	0	0.09	0.06
*Kraussaria angulifera*	0	0.09	0.06	0.1	0	0.16	0	0	0.03	0	0	0
*Acrida bicolor*	0	0.11	0.09	0.08	0	0.21	0	0	0.12	0	0.13	0
Imipenem	0.09	0.07	0.31	0.19	0.07	0.15	0.16	0.19	0.11	0.09	0.06	0.09
Index of multi-resistance to insects’ extracts (IMRE)												
Values of IMRE	0.85	0.15	0.15	0.15	0.85	0.23	0.46	0.46	0.46	0.61	0.30	0.46

## Correlations between bacteria and insect extracts

**Figure 3:**
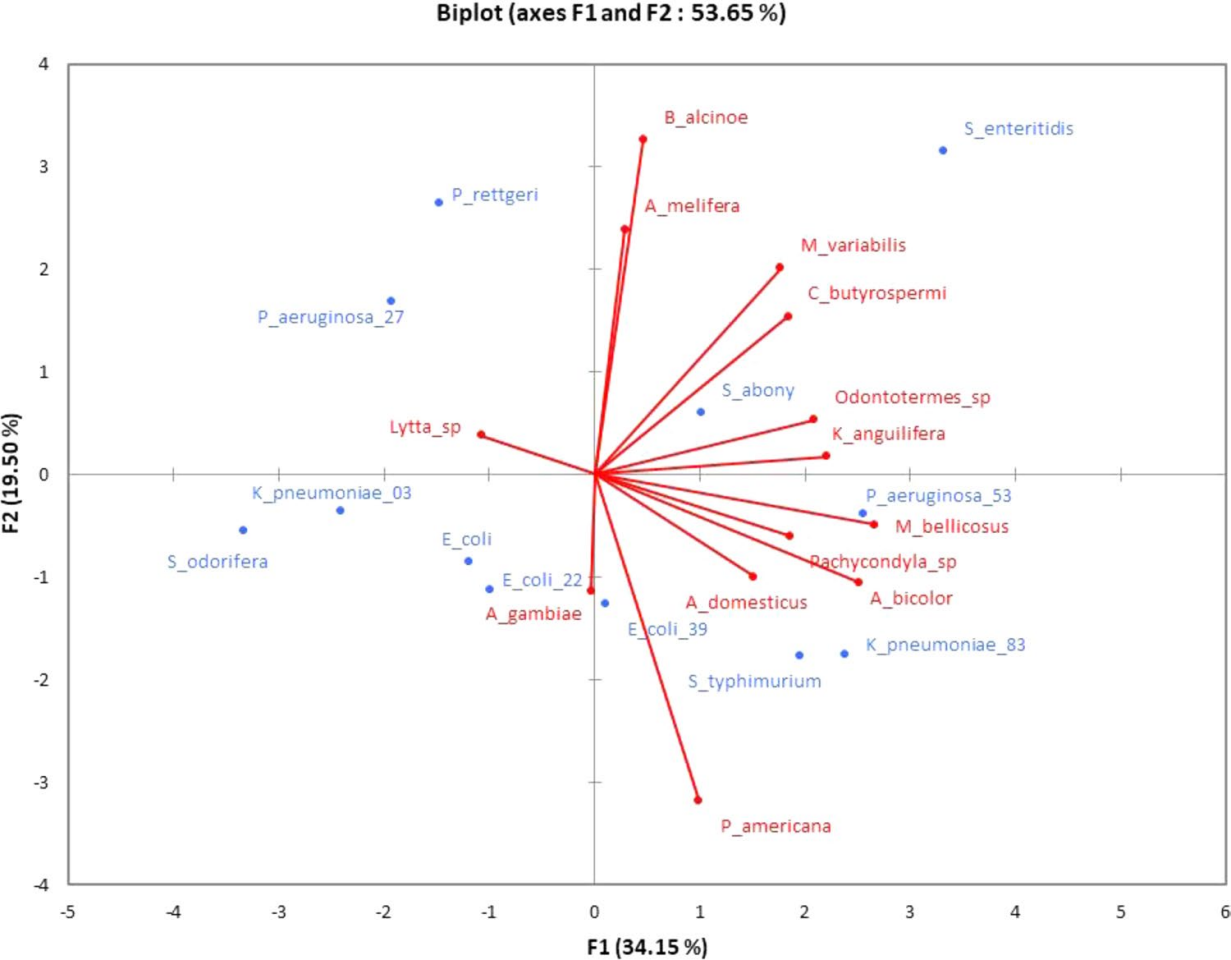
PCA of correlation between growth inhibitor extracts and bacteria.

Principal Compound Analysis (PCA) was performed to understand the interaction between the extracts of the different insects and the Gram-bacteria used in the different tests (Fig. 3). According to the first two axes (F1 and F2), which account for 53.63% of the dispersion of the results, a strong positive correlation appears between the two insect extracts and *Providencia rettgeri* 652655. These extracts are from *Bunaea alcinoe* and *Apis mellifera*. Therefore, these extracts are indicated for inhibiting the proliferation of the aforementioned bacteria. However, it should be noted that *Providencia rettgeri* 652655 is very sensitive to *Apis mellifera* extracts. Four insects, *Bunaea alcinoe*, *Apis melifera*, *Myrabris variabilis*, and *Cirina butyrospermi*, also have a positive correlation with *Salmonella enteritidis* ATCC13076. These extracts could be used to prevent the proliferation of these bacteria and to avoid nuisances due to the last debt. The extracts of *Bunaea alcinoe* and *Myrabris variabilis* were more correlated with *Salmonella enteritidis* ATCC13076. Therefore, they would be the best inhibitors of this bacterium. From the above, *Bunaea alcinoe* and *Apis mellifera* extracts could be used for both *Salmonella enteritidis* ATCC13076 and *Providencia rettgeri* 652655 inhibitions. In contrast, the extract of *Periplaneta americana* represents an extract that can be effective simultaneously against the growth of *Klebsiella pneumoniae* ATCC13883, and *Salmonella* Typhimurium ATCC14028.

## Conclusion

This study made it possible to highlight the antibacterial activity of insect extracts against gram-negative bacteria. The bacteria tested in this study are responsible for several pathologies constituting a major health problem in Burkina Faso. Entomotherapy can be an alternative treatment for certain pathologies in Burkina Faso. However, this opportunity is rarely exploited in Burkina Faso, which is full of a wide variety of insects.

## Data Availability

Data included in article/referenced in article.
